# The Clinical Significance of MicroRNAs in Colorectal Cancer Signaling Pathways: A Review

**DOI:** 10.1055/s-0043-1777094

**Published:** 2023-11-22

**Authors:** Athanasios Michas, Vasileios Michas, Evangelos Anagnostou, Michail Galanopoulos, Maria Tolia, Nikolaos Tsoukalas

**Affiliations:** 1 Department of Oncology, 401 General Military Hospital of Athens, Athens, Greece; 2 Department of Radiology, Achepa General Hospital Thessaloniki, Thessaloniki, Greece; 3 Department of Neurosurgery, Queen Elizabeth Hospital Birmingham, Birmingham, United Kingdom

**Keywords:** microRNAs, genomics, colorectal cancer, molecular pathways

## Abstract

Colorectal carcinoma (colon and rectum) is currently considered among the most prevalent malignancies of Western societies. The pathogenesis and etiological mechanisms underlying colorectal cancer (CRC) development remain complex and heterogeneous. The homeostasis and function of normal human intestinal cells is highly regulated by microRNAs. Therefore, it is not surprising that mutations and inactivation of these molecules appear to be linked with progression of colorectal tumors. Recent studies have reported significant alterations of microRNA expression in adenomas and CRCs compared with adjacent normal tissues. This observed deviation has been proposed to correlate with the progression and survival of disease as well as with choice of optimal treatment and drug resistance. MicroRNAs can adopt either oncogenic or tumor-suppressive roles during regulation of pathways that drive carcinogenesis. Typically, oncogenic microRNAs termed oncomirs, target and silence endogenous tumor-suppressor genes. On the other hand, tumor-suppressive microRNAs are critical in downregulating genes associated with cell growth and malignant capabilities. By extensively evaluating robust studies, we have emphasized and distinguished a discrete set of microRNAs that can modulate tumor progression by silencing specific driver genes crucial in signaling pathways including Wnt/b-catenin, epidermal growth factor receptor, P53, mismatch repair DNA repair, and transforming-growth factor beta.

## Introduction

Colorectal carcinoma (colon and rectum) is one of the most prevalent malignancies of Western societies. More specifically, the proportion of colorectal cancer (CRC) accounts for approximately 8 to 9% of newly diagnosed cancer cases in the United States, comprising the second leading cause of cancer-related deaths annually. Recently, the development of effective treatment and screening strategies have significantly contributed to a steady decline in incidence and disease-specific mortality. Nevertheless, increasing rates of disease in younger adults and the exponential increases of cases in economically emerging countries such as Eastern Europe and Asia remain a significant concern. 1

The pathogenesis and etiologic mechanisms underlying the development of CRC remain complex and heterogeneous. A variety of environmental factors appear to contribute to the development of these tumors. Nevertheless, although CRC appears to be largely influenced by lifestyle factors, it is evident that interindividual genetic variation significantly affects the prevalence and severity of disease. 2 Eventually, over the last decades scientists have finally succeeded in linking CRC to genetic predisposition in certain family trees. Accordingly, they identified specific critical mutations that influence the tumorigenesis of CRC.

Actually, only 15 to 20% of CRCs show clear hereditary predisposition. The vast majority of colorectal tumors occurs sporadically and usually affects individuals without a family history or genetic predisposition. Although familial cases represent only a small proportion, they have provided key insights into the molecular pathways of disease. As several studies have shown, sporadic CRCs share the same genetic abnormalities as their inherited counterparts. In general, sporadic colorectal tumors exploit similar genetic mechanisms of proliferation as hereditary cancers but arise from driver mutations occurred in somatic cells. For example, APC gene, which has prominent role in the biology of FAP syndrome, also found to have somatic mutations in approximately 50 to 55% of sporadic CRC cases. 3 These mutations influence the progressive acquisition of genetic and epigenetic alterations that eventually lead to malignancy ( Table 1 ). 4

**Table 1:** Percentage of sporadic, familial, and inherited cases of colorectal cancer 4

Sporadic cases	65%	
Hereditary cases	High-penetrance genes (APC, BRCA, MLH1, MSH2, MSH6, TP53, etc.)	5%
	Moderate-penetrance genes (MUTYH, CHEK2, ATM)	5%
Familial cases	25%	

The most extensively studied sequences of the human genome are those of protein-coding genes. 5 The Central Dogma of Biology, established by Fransis Crick in 1970, dictates that DNA is transcribed into RNA, which is subsequently translated, and that proteins constitute the major players of biological mechanisms. 6 However, after the publication of the Human Genome Project in 2003, the perspective of genomic research has significantly amended. During the last two decades the development and widespread implementation of innovative methods in the field of genomics led to the discovery that only 1.5 to 2% of human genome encodes for functional peptides. 7 Eventually, it was revealed that our genome encodes approximately 20,000 to 25,000 protein genes, similarly with much simpler organisms such as the fruit fly. 8 Therefore, it is not surprising that the majority of single-nucleotide polymorphisms, identified to correlate with human malignancies including colorectal tumors through large genome-wide association studies, are located in large introns or distal to coding regions, an area previously been considered as unexplored territory. 9 More recently, the advent of next-generation sequencing and multiomics analyses have led to projects like ENCODE providing unequivocal evidence that nearly 90% of the human genome is dynamically and pervasively transcribed in noncoding RNAs. 10 11

Since then, a substantial amount of research has been devoted to the identification and interpretation of these noncoding RNAs. The emerging landscape indicates that noncoding transcripts represent a broad and heterogeneous group of molecules with diverse functions and key regulatory roles in various biological processes. 12 Several studies show that noncoding RNAs utilize a variety of mechanisms and participate in fundamental cellular activities such as proliferation, differentiation, migration, and apoptosis, through transcriptional and posttranscriptional regulation of gene expression. 13

High-throughput sequencing techniques and microarray analyses have identified thousands noncoding RNAs in the human genome. Clearly, the presence of a highly sophisticated regulatory network, interacting with the protein-coding genome (Exome), has a fundamental impact in human pathophysiology with significant biological and biomedical consequences. Currently, it is evident that noncoding RNAs influence almost all genomic processes and biological pathways of eukaryotic organisms. Therefore, obviously as CRC constitutes a genomic disease, mutations, and aberrant expression of noncoding RNAs have come to surface as hallmark of tumorigenesis. Accordingly, a plethora of important studies have revealed significant association between noncoding genomic regions and pathogenesis of intestinal malignancies.

Two decades ago both the existence and the importance of these molecules remained unknown. Their detection was achieved by the collaboration of Ambros's and Ruvkun's laboratories, through experiments in the nematode *Caenorhabditis elegans* , with the discovery of the lin-4 microRNA. 14 Since then, scientific research has elucidated their mechanisms of biogenesis, modes of action, and biological roles. MicroRNAs are small single-stranded RNA transcripts with 18 to 25 nucleotides length that comprise vital part of the genome in the majority of eukaryote organisms. 15 Generally, they are well-characterized regulatory noncoding RNAs, which account approximately 1 to 2% of human genome in total. As plethora of scientific studies support, they represent a conserved family of small RNA molecules with extremely important role in the posttranscriptional regulation of gene expression. MicroRNAs are transcribed as long precursor molecules that undergo sequential multistep processing to produce mature single-stranded RNAs that manipulate the expression of other genes. 16 Regarding location, microRNAs are found through the whole range of human genome. They often reside not only in intergenic (desert) regions, but also in introns of coding transcripts, although some may even overlap with exonic sequences. 17 In addition, a large amount of microRNAs are organized in clusters and transcribed as polycistronic units. 18 CRC progression involves several mechanisms, including most notably overproliferation, defects in apoptotic regulation, increased angiogenesis, and acquisition of invasive and metastatic phenotype. 19 Since the first association between microRNA expression and CRC, made by Michael et al in 2003 who found decreased levels of miR-143 and miR-145 in tumor tissues compared with healthy adjacent, huge amount of research has been dedicated to unravel the molecular effects of these tiny molecules. 20 21

## Methods

To elucidate the influential role of microRNAs on the major molecular signaling pathways that drive CRC tumorigenesis, we performed in-depth review and analysis of the robust and valid literature. The terms used were “microRNAs or mirs,” “colorectal cancer,” and “molecular signaling pathways.” Up to date (October 2023), totally 1,994 studies were retrieved in PubMed, Medline, and Queen Mary University of London official library, in which approximately 40 concerned clinical trials investigating microRNA differential expression patterns in CRC tissue specimen and plasma samples. By extensively evaluating these studies, we have emphasized and distinguished a discrete set of microRNAs that can modulate tumor progression by silencing specific driver genes crucial in signaling pathways like Wnt/b-catenin, epidermal growth factor receptor (EGFR), P53, mismatch repair DNA repair, and transforming-growth factor Beta (TGF-b).

## Discussion

In general, CRC was thought correlated with a comparative decline in the total microRNA expression. 22 Studies have shown that in the context of global microRNA depletion, the defects of tumor-suppressor microRNAs have greater effects on driving tumorigenesis than depletion of oncogenetic microRNAs (oncomirs). This phenomenon was further supported by animal-based models that revealed Dicer1, which is key enzyme of physiological microRNA biogenesis, as a major tumor-suppressor gene in CRC cell lines. 23 Nevertheless, that issue still remains ambiguous and highly controversial. 24 Interestingly, a work by Wang et al found the majority of microRNA expression to be globally elevated in CRC. 25 Converging with these findings, more recent studies of microRNA profiles in CRC indicated that approximately two-third of altered microRNAs in CRC have shown enhanced expression. Authors concluded that microRNA processing machinery probably is not compromised in colorectal malignancies. 26 These conflicting results suggest that microRNAs should be independently evaluated between diverse tumor types and patients.

### Wnt/b-Catenin Signaling Pathway

Aberrant and hyperactivated Wnt/b-catenin signaling is a major mechanism of colorectal tumorigenesis and early-cancer progression. Therefore, microRNAs that regulate this pathway play important role in maintaining the physiology of normal colonic cells. 27 An increasing amount of studies have identified specific microRNA mutations that influence Wnt/b-catenin cascade, either by direct suppression of APC gene, or indirectly by targeting alternative pathway components. 28 Mir-135 is a well-studied molecule that directly targets the 3′ UTR of APC gene in human colorectal cells. Therefore, enhanced Mir-135 family expression implies upregulation of Wnt pathway and is associated with advanced tumor grade and poor clinical outcome. 29 30 Colonic cancer progression via immediate inhibition of APC gene is also clear and extensively validated for miR-494 and miR-19. 31 32 In addition, mir-21, which is among the most famous oncomirs, shows positive expressional correlation with key components of Wnt pathway including b-catenin and cyclin D1. This observation is modulated through repression of multiple mir-21 targets, most of which are genes that control cell-cycle like PTEN. 33 Moreover, animal model studies have shown that enhanced expression of mir-574–5p promotes colon cancer progression. This molecule activates Wnt stimulation, via downregulating Quaking 6/7 RNA-binding protein that controls several intracellular processes including proliferation, differentiation, and angiogenesis. 34 To further support the critical role of stimulatory Wnt/b-catenin oncogenic microRNAs, Li et al demonstrated that mir-224 targets and inhibits the GSK3b and secreted Frizzled Related Protein 2, significant suppressors of this precise signaling pathway. Consequently, results in increased nuclear translocation of b-catenin thereby enhanced expression of target genes c-Myc and cyclin D1. 35 Furthermore, mir-145 is a tumor-suppressor agent that normally inspects and moderates the intracellular translocation of b-catenin into the nucleus, crucial step for signaling activation. Therefore, truncating mutations and reduced expression are typical events in colorectal tumors. 36 Moreover, mir-28–5b mechanistically attenuates Wnt signaling, by suppressing a calmodulin-binding transcription activator termed CAMTA2. As revealed by a contemporary research, CAMTA2 is significantly upregulated in many colorectal tumors and associates with poor survival. Authors concluded that mir-28–5b/CAMTA2 axis is critical in CRC development and might be a promising diagnostic and therapeutic marker too. 37 Similarly, mir-93 significantly decreases Wnt pathway activation, through targeting Smad-7, which is essential for nuclear accumulation of b-catenin. 38 Consistent with these findings, Zhang et al also recognized mir-7 as a strong tumor-suppressor gene for colorectal tumorigenesis, through downregulation of YY1 oncogene in normal colonic cells. 39 By applying in silico searches, luciferase reporter assays, and western blot analyses, researchers identified an evolutionary-conserved mir-7 binding site in the 3′UTR of YY1. This gene exerts broadly oncogenic functions through Wnt signaling pathway by activating b-catenin, antiapoptotic survivin, and fibroblast growth factor 4. Furthermore, a very recent report identified the significant implication of mir-103/107 in colon tumorigenesis. This molecule stimulates stem-like features in CRC cells including self-renewal, angiogenesis, and chemoresistance by directly repressing Axin 2, which is a strong inhibitor of Wnt/b-catenin signaling cataract. 40

### Epidermal Growth Factor Receptor Signaling Pathway

EGFR is a transmembrane glycoprotein, member of the human EGFR family. When activated, it promotes intracellular signaling via stimulation of two major subnetworks, KRAS/RAF/MEK and PI3K/AKT, respectively. It constitutes a well-characterized pathway with critical role in the survival, proliferation, migration, angiogenesis, and apoptosis of human cells, involved in several types of epithelial cancers including CRC. 41 A series of investigations in the field revealed the extensive implication of microRNAs in regulation of EGFR signaling that significantly enhanced our detailed understanding of intestinal carcinogenesis. 42 Two of the most important EGFR-related microRNA tumor-suppressor genes are mir-143 and mir-145. Expression analyses have shown that these molecules are significantly diminished in cells of colorectal tumor origin compared with normal epithelium. Among their gene targets are several members of the EGFR pathway, notably KRAS and BRAF. 43 As identified, mir-143 and mir-145 orchestrate a well-coordinated program of gene repression, where either they share a target transcript, or both their target transcripts converge in a common signaling cascade. 44 In addition, Let-7 microRNA is a well-known tumor-suppressor molecule that regulates EGFR. Let-7 directly targets KRAS mRNA in a variety of tumors including lung and breast cancer. 45 However, surprisingly, an analysis showed that Let-7 provided increased expression in metastatic CRC tissues compared with normal mucosa, but only in KRAS-mutated tumors. 46 Researchers concluded that this microRNA could be applied as a distinctive biomarker regarding treatment with anti-EGFR targeted therapy like cetuximab. Moreover, recent studies have acknowledged several novel suppressor microRNAs associated with EGFR cataract. For example, mir-19, mir-217, and mir-181d can inhibit angiogenesis, proliferation, and invasion of colorectal tumors by interacting with downstream regulators like PEAK1. 47 48 49 On the other hand, mir-31 appears to be a potent stimulator of KRAS in colorectal tumors, through negative regulation of RASA1, which is inhibitor of KRAS function. 50 Furthermore, mir-210 and mir-181a constitute closely related with KRAS activation molecules, as significant oncomirs that promote CRC progression and invasiveness. 51

At the same time, AKT-PI3K-mTOR/PTEN represents the second signaling hub of the EGFR pathway, being amplified in almost 20% of CRCs. 52 Multiple oncomirs and tumor-suppressor microRNAs interact and control functioning of this cascade under both physiological circumstances and malignancy. For instance, miR-17–92 cluster, also known as oncomir-1, directly targets 3′UTR and inhibits PTEN gene, driving aberrant cell proliferation and atypical angiogenesis. 53 Furthermore, mir-21, mir-19, and mir-96 are important oncogenes also found to be involved in CRC carcinogenesis through stimulation of AKT/PIK3 pathway. 54 In addition, mir-126 is a significant tumor-suppressor gene with crucial implications in the control of EGFR signaling. This molecule specifically silences p85b protein-coding gene, which is necessary substrate regarding the stability and propagation of PIK3 network. 55 Interestingly, mir-126 also activates VEGF-induced angiogenesis by modulating expression of PIK3R2 (PI3K regulatory subunit 2). 56 Consequently, abrogation of mir-126 potentially provides a selective growth advantage during tumorigenesis. Moreover, through animal model experiments, mir-497 was also identified to exhibit antiproliferative and antiapoptotic action by targeting VEGF receptor 2 in colonic tissues. 57 Furthermore, a very recently published study revealed the significant involvement of mir-27b in the control of PIK3CA signaling cascade. 58 Overexpression of this molecule inhibits colorectal tumor cells proliferation and migration, via suppressing PI3K p110a subunit. Researchers concluded that mir-27b could be tested as potential therapeutic antitumor agent for colorectal malignancies.

### P53 Signaling Pathway

Tumor-suppressor gene P53 is one of the most frequently mutated genomic regions in human malignancies. It is known as ‘the guardian of the genome’ gene, owing to the numerous fundamental roles in maintaining the cellular physiology under diverse stress conditions. 59 Specifically, more than 50% of CRCs display of some degree inactivation in p53 function. 60 Several microRNAs have been recognized recently as significant components of p53 signaling cascade across these tumors. 61 Contemporary scientific knowledge suggests that albeit P53 regulates transcription and maturation of multiple downstream tiny RNAs by activation or repression of distinct molecules, vice versa, TP53 expression is also under the tight control of particular microRNAs. 62 For instance, Shi et al reported that P53 can induce miR-15a/16–1 to form a double-negative feedback loop with transcription factor AP4. 63 Then, activated AP4 performs critical function regarding invasiveness and metastasis of colorectal malignancies. In addition, in silico searches identified miR-504 as a novel microRNA that can negatively regulate p53 expression via two binding sites in the human p53–3′UTR. 64 Ectopic expression of this molecule impaired p53 protein levels and function, especially in mediating apoptosis and G1 cell cycle arrest. As experimentally revealed, mir-504 overexpression promotes colon cancer tumorigenicity in vivo. 65 Actually, translational repression of TP53 is controlled by a wide variety of microRNAs including mir-125b, mir-25, mir122, mir-30d, and mir-518c. 64 MicroRNA-125b is a brain-enriched tiny RNA that acts as negative regulator of p53 in both zebrafish and humans. 66 In vitro overexpression of Mir-125b reduces endogenous levels of p53 protein and impairs physiological apoptosis during development and stress response. Moreover, mir-25 and mir-30 compromise tumor-suppressive functions, via negative regulation of both gene expression and protein level of p53. 67 Interestingly, mir-518c can simultaneously target and inactivate both p53 and PTEN genes. 68 The huge intricacy of p53 signaling pathway was further illustrated through studies around mir-34a. This molecule is a downstream transcription target of p53 protein during apoptosis but concomitantly was found to enhance p53 expression through direct negative regulation of SIRT1 that physically interacts and disrupts the protein. 69 Additionally, p53 induces and manipulates the expression of several supplementary microRNA molecules, to enhance tumor suppression. These include mir-107, mir215, mir-143, mir-145, and mir-192. 64 70 71 72 Such findings demonstrate the huge importance of microRNAs expression and interactions, to mediate the proper function of p53 tumor-suppressive signaling cascade.

### Mismatch Repair Machinery

Microsatellite instability (MSI) is a hallmark feature of defective mismatch repair (MMR) system machinery. Lynch syndrome is a well-known syndrome related to germline defective mutations of MMR system. This hypermutagenic phenomenon is present in approximately 15% of sporadic CRC cases. Although, the microRNA profiles of CRC have been studied extensively, comparatively few analyses have specifically investigated microRNA signatures in the presence of MSI. 73 Nevertheless, certain microRNAs have recently emerged as significant regulators of genes associated with MMR, including MLH1, MSH2, MSH6, and PMS2. 74 Valeri et al identified that mir-155 compromises MMR mission by targeting the 3′UTR of MLH1, MSH2, and MSH6. 75 Overexpression of this molecule correlated with a hypermutated phenotype and MSI in CRC cell lines. In addition, mir-21 downregulates the MMR recognition complex hMutSa, by interacting with the two core gene subunits MSH2 and MSH6. 76 Colorectal tumors overexpressing mir-21 showed MSI and also displayed reduced response to 5-fluorouracil-based chemotherapy. 77 Therefore, mir-21 could be further evaluated as treatment response biomarker. Moreover, Sarver et al identified a small panel of six microRNAs that could successfully distinguish MSI from microsatellite stable colon tumors. 78 Among them, mir-31 and mir-625 were overexpressed in MSI malignancies, whereas mir-552, mir-592, mir-181c, and mir-196b performed decreased expression in stable cancers. More interestingly, microRNA patterns could be applied to distinguish hereditary and sporadic colorectal tumors with MSI. Although these two conditions share the same molecular mechanism of tumor development, the underlying cause is quite different. Balaguer et al applied microarray analysis data, to reveal a group of 31 microRNA molecules that could be used as classifiers with high accuracy (area under the receiver operating characteristic curve: 0, 94). 79 Consequently, microRNAs prove to have fundamental roles for the diligent function of DNA damage/repair machinery, especially in the terms of colorectal carcinogenesis.

### TGF-b Signaling Pathway

TGF-b/Smad is an important molecular pathway involved in cell proliferation and associated with cancer invasiveness and metastatic potential. Regarding CRC, this pathway performs dual functions as it is a tumor-suppressor at the early stages but simultaneously acts as a powerful cancer promoter in advanced neoplasms. 80 Several microRNAs have been identified to interact and regulate the TGFB2 receptor (TGFBR2), which is the key component of initiating signaling. 81 For instance, mir-135 is a well-studied molecule, confirmed by many researchers to be significantly elevated in CRC cells. 82 As recently revealed mir-135 directly disturbs TGFBR2 translation and consequently promotes cell proliferation and inhibits apoptosis. 83 Similarly, mir-301a also targets TGFRB2 and is correlated with increased tumor aggressiveness and metastatic dissemination. 84 85 To further support the critical role of TGFRB2, studies have shown that the prominent mir-21 oncogene also regulates that critical receptor via direct binding to the 3′UTR. 86 Moreover, Smad-4 is an important mediator of TGF-b signaling cascade, whose abrogation results in distal metastases and generally poor prognosis. MicroRNA-20–5p and mir-224 significantly silence Smad gene and induce proliferation and cancer invasiveness. 87 88 Furthermore, mir-1269 is a recently well-studied molecule, usually found increased in advanced stage cancer tissues. TGF-b activates mir-1269, whereas vice versa mir-1269 enhances TGF-b signaling by targeting Smad-4, hence forming a positive feedback loop. 89 This molecule is associated with treatment relapse and metastasis, suggesting a potential useful biomarker regarding choice of adjuvant chemotherapy.

**Fig. 1:**
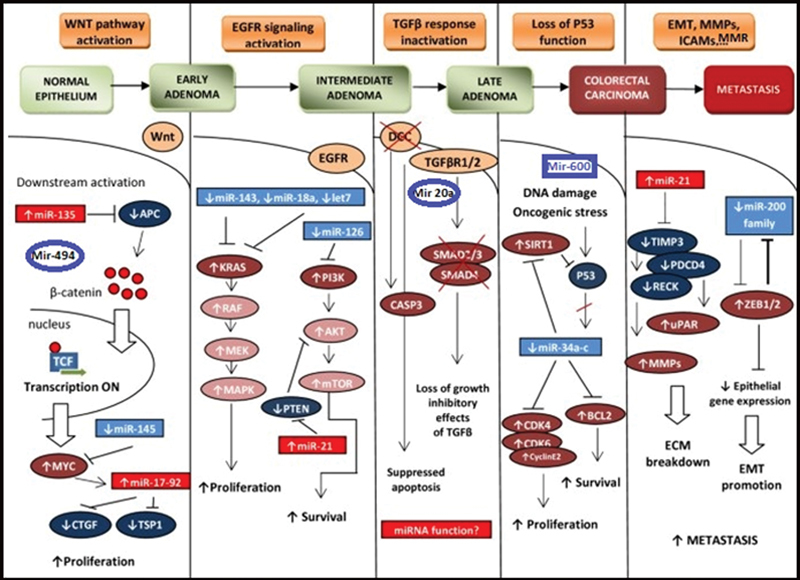
A proposed graphic scheme of miRNAs impact on development of colorectal cancer. EGFR, epidermal growth factor receptor; MMR, mismatch repair.

A summary of distinct microRNA molecules and their respective gene targets and biological effect is illustrated in Table 2 ( Fig. 1 ).

**Table 2:** microRNA molecules and their respective gene targets and biological effect

MicroRNAs with respective mRNA targets associated with colorectal cancer				
Key signaling pathway	microRNA	Targets	Target effects	References
Wnt/b-catenin pathway	Mir-135	APC	Proliferation	90
	Mir-21	PTEN	Progression/invasion/metastasis	91
	Mir-145	b-catenin	Proliferation/migration	92
	Mir 103/107	Axin 2	Angiogenesis/chemoresistance	93
	MIR-494	b-catenin	Progression/metastasis	94
	TrkC-miR2	TrkC	Proliferation	95
EGFR pathway	MIR-143	BRAF	Proliferation/angiogenesis	96
	LET-7	KRAS	Progression/invasion/metastasis	97
	Mir-210	KRAS	Progression/invasion/metastasis	98
	Mir-217	KRAS/MAPK1	Proliferation/angiogenesis	98
	Mir-19	KRAS, VEGF	Proliferation/angiogenesis	99
TP53 pathway	MiR-339–5p	MDM2 gene	Proliferation/invasion	100
	MiR-34a	c-Kit gene	Proliferation	101
	MiR-600	TP53 gene	Proliferation/invasion	102
MMR	Mir-155	MLH1 gene	Microsatellite instability	103
	Mir-625	MMR	Microsatellite instability	104
TGF-b	MiR-20a	TGF-b gene	Proliferation/invasion	105
	miR-20a/b	Bcl-2	Apoptosis	106

Abbreviations: EGFR, epidermal growth factor receptor; MMR, mismatch repair.

## Conclusion

CRC is one of the most commonly diagnosed malignancies worldwide, with considerable morbidity and mortality. The recent and revolutionary advancements in comprehension of disease biology have significantly elucidated the diverse nature and pathogenesis of these tumors, promising a more favorable future for patients and doctors. Noncoding genomic regions and most notably noncoding RNAs appear to have crucial functional implication in both physiological and pathological processes of colorectal cells. Somatic genetic variations of loci without protein coding potential have been detected to contribute to the progressive transformation of normal colonic mucosa to adenocarcinoma, through regulation of several fundamental signaling pathways involved in chromosome instability, MSI, and serrated neoplasia cascades. As demonstrated through in-depth investigation of numerous robust studies, colorectal malignant cells possess a variety of mutational alterations and express abnormally several noncoding genomic regions that influence important signaling cascades toward carcinogenesis. Thus, identification and detailed interpretation of noncoding driver mutations may enable effective screening and risk assessment as well as individualized therapeutic approaches targeting specific proteins. The huge scientific interest around regulatory genome and especially noncoding RNAs promises that the upcoming years will be marked by the development of noncoding RNAs-based therapy and establishment of a contextual role next to current diagnostic, preventive, and treatment strategies of colorectal tumors.
